# Physician antibiotic hydration preferences for biologic antibacterial envelopes during cardiac implantable device procedures

**DOI:** 10.3389/fcvm.2022.1006091

**Published:** 2022-12-22

**Authors:** Thomas F. Deering, John N. Catanzaro, David A. Woodard

**Affiliations:** ^1^Department of Cardiology, Piedmont Heart Institute, Atlanta, GA, United States; ^2^Division of Cardiology, Department of Medicine, UF Health Cardiovascular Center, Jacksonville, FL, United States

**Keywords:** cardiovascular implantable electronic device (CIED), defibrillator, envelope, antibacterial envelope, extracellular matrix, ICD (implantable cardioverter-defibrillator), infection, pacemaker

## Abstract

**Background:**

Cardiac implantable electronic device (CIED) infection is a potentially serious complication of CIED procedures. Infection risk mitigation includes using guideline-recommended pre-operative intravenous antibacterial prophylaxis (IV ABX). The use of antibiotic-eluting CIED envelopes has also been shown to reduce infection risk. The relationship between and potential benefits associated with guideline-recommended IV ABX in combination with antibacterial envelopes have not been characterized.

**Methods:**

Biologic envelopes made from non-crosslinked extracellular matrix (ECM) were implanted into 1,102 patients receiving CIEDs. The implanting physician decided patient selection for using a biologic envelope and envelope hydration solution. Observational data was analyzed on IV ABX utilization rates, antibacterial envelope usage, and infection outcomes.

**Results:**

Overall compliance with IV ABX was 96.6%, and most patients received a biologic envelope hydrated in antibiotics (77.1%). After a mean follow-up of 223 days, infection rates were higher for sites using IV ABX <80% of the time vs. sites using ≥80% (5.6% vs. 0.8%, *p* = 0.008). Physicians demonstrated preference for hydration solutions containing gentamicin in higher-risk patients, which was found by multivariate analysis to be associated with a threefold reduction in infection risk (OR 3.0, 95% CI, 1.0–10.0).

**Conclusion:**

These findings suggest that use of antibiotics, particularly gentamicin, in biologic envelope hydration solution may reduce infection risk, and use of antibacterial envelopes without adjunct IV ABX may not be sufficient to reduce CIED infections.

**Clinical trial registration:**

[https://clinicaltrials.gov/], identifier [NCT02530970].

## Introduction

Cardiac implantable electronic devices (CIEDs) are important tools in the management of patients with a variety of arrhythmia and heart failure disorders. Expanding device indications, newer CIED technology, and population demographics have resulted in continuous growth in the use of CIEDs (1). This growth in volume has been associated with higher complication rates including infection. Reported rates of CIED infections range from approximately 1–3% for *de novo* implantations with reported rates up to 7% following device replacements and higher rates observed among patients with risk factors that have been associated with CIED infection (2–5). Several factors have been postulated as influencing CIED infection rates, which exceed overall CIED implantation growth rates, including an aging demographic profile, the presence of more comorbidities, and a greater prevalence of infection risk factors among recipients (3, 5–13).

Cardiac implantable electronic device infections are associated with substantial morbidity and mortality that augment healthcare costs (1, 2). Based upon information obtained from the National Inpatient Sample database, which is the largest all-payor US-based inpatient database, the mortality rate due to lead extraction was 4.5%, with higher mortality rates in patients >85 years old (5.3%) when compared to those aged 18–44 years (2.5%, *p* < 0.001) (14). Furthermore, the mortality rate among patients undergoing lead extraction associated with an infection was four times higher than rates observed among patients undergoing extraction for another reason. This analysis also demonstrated that there was a 53% increase in the number of hospitalizations due to CIED infections, a doubling in the percentage of lead extractions secondary to infection (14–29%), and a 41% overall increase in total number of extractions between 2003 and 2011. The economic impact associated with CIED infection was also substantial with a 53% increase in mean hospitalization charges ($91,348–$173,211, *p* < 0.001). Retrospective studies of commercial and Medicare databases have reported mean payments for the management of CIED infections ranging from $22,856 to $77,397 per patient with average adjusted annual medical costs 2.4 times greater for patients with a CIED infection (15, 16).

Based on these clinical and economic concerns, infection prevention is a key consideration associated with the implantation of cardiac devices. Evidence-based prophylactic approaches include the utilization of guideline-directed preoperative intravenous antibacterial prophylaxis (IV ABX), the use of strict skin antisepsis, and potentially the employment of antibacterial CIED envelopes (1, 17–19). In the large Prevention of Arrhythmia Device Infection Trial (PADIT), there was no difference in the CIED infection rate between patients receiving a conventional perioperative antibiotic regimen vs. those undergoing an incremental antibiotic approach (19). The 0.9% hospitalization-for-infection rate in the overall population and 1.11% rate in the high-risk group establish standards for best-in-class infection rates, especially given the large and geographically diverse enrollment (19,603 patients from 28 centers) (19).

**FIGURE 1:**
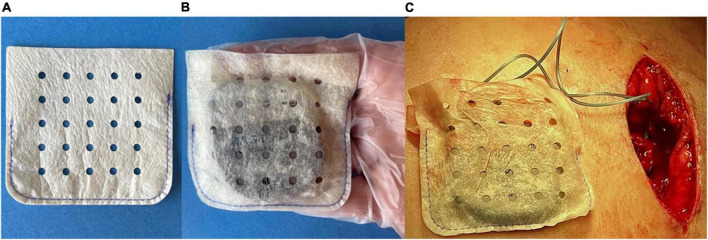
**(A)** A medium-size biologic CanGaroo^®^ Envelope made from 4-ply lyophilized, decellularized, non-crosslinked extracellular matrix derived from porcine intestinal submucosa (SIS ECM). **(B)** Once hydrated by the implanting physician per manufacturer instructions, the biologic envelope handles well and conforms to the device. **(C)** Intraoperative photo of the hydrated biologic envelope just prior to being placed within the tissue pocket.

Two types of CIED envelopes that are designed to stabilize the CIED within the pocket are available for use in the US. The biologic envelope is made from a decellularized, non-crosslinked extracellular matrix derived from porcine intestinal submucosa (SIS ECM) (Figures 1A–C) (20). The non-biologic envelope consists of an absorbable multifilament block copolymer coated with an absorbable polyarylate polymer containing the drug substances rifampin and minocycline (20).

The use of non-biologic envelopes has been associated with a reduced incidence of CIED infections in high-risk patients in a controlled trial (21). Published studies suggest that non-biologic surgical materials potentially can trigger a robust foreign body response, including chronic inflammation and fibrous encapsulation of the material, rather than fostering integration into host tissues (22–25). However, it remains uncertain whether non-biologic products employed in a standard surgical arena generate the same tissue response as those products employed as a CIED envelope. In contrast, materials made from biologic non-crosslinked ECM have been shown to foster greater tissue integration and vascular ingrowth, a modulated inflammatory response, and rapid clearance of bacteria (22, 23, 25–31).

Biologic envelopes are hydrated prior to use by the implanting physician, who may elect to add antibiotics to the hydration solution to augment local antibiotic levels and enhance the inherent antimicrobial properties of the ECM. Once implanted, growth factors released from the ECM stimulate angiogenesis and allow host immune cells to penetrate the remodeling envelope (32–34). The immunomodulatory process that ECM triggers during remodeling leads to decreased scar tissue formation and a vascularized capsule, fostering the development of a healthy long-term pocket (23, 25, 33, 34). ECM-based materials have also been shown to natively promote the removal of bacteria after implantation, and compounds with inherent antimicrobial properties are released during remodeling, potentially mitigating infection risk (29–31, 34).

The CanGaroo^®^ Envelope (Aziyo Biologics, Inc., Silver Spring, MD, USA) is a biologic ECM envelope constructed of 4-ply decellularized, non-crosslinked, lyophilized SIS ECM. Recently published data suggest that the CanGaroo Envelope can reduce the risk of device migration and erosion and may facilitate device removal, when future exchange or revision is required, due to reduced scar formation, encapsulation, and foreign body response (35, 36).

In this manuscript, we combined data from two post-market observational studies (SECURE and CARE) with the aim of evaluating differences in real-world clinical decision making with regard to the use of antibiotic solutions for rehydration of the biologic envelope and the use of IV ABX when the envelope was implanted in a broad population of patients undergoing implantation of a CIED.

## Materials and methods

### Study design

This report includes findings from 2 studies. SECURE (NCT02530970) was a prospective, multicenter, post-market, observational study conducted at 39 sites in the United States between September 2015 and November 2017. CARE was a retrospective, post-market observational study conducted at Piedmont Athens Regional Hospital in Athens, GA, USA of patients who received treatment between August 2014 and July 2015. Both studies were designed to evaluate clinical outcomes following the use of the CanGaroo Envelope in patients who underwent implantation of a CIED. Patients were eligible for enrollment if they received a CanGaroo Envelope at the time of their CIED implantation. Patient selection for receiving an envelope was left to the discretion of the participating physicians.

All patients underwent a comprehensive medical history and clinical examination prior to implantation. Study protocols were approved by the Institutional Review Board of each participating center, and all patients provided informed consent. The studies were conducted in accordance with the Declaration of Helsinki and regulatory and institutional requirements.

### Surgical technique

Cardiac implantable electronic device implantation was performed according to standard techniques. The envelope size was selected by the investigator, based on the size of the CIED being implanted. In both studies, the envelope hydration solution and choice of antibiotics, if used, were left to individual physician discretion.

The CIEDs were connected to the leads and the leads were secured to the underlying tissue before the CIED was placed into the envelope and subsequently implanted. The pre- and post-procedure medication regimens as well as clinical treatment were performed according to the routine practice of each center.

### Data collected

Patient, procedural, and follow-up data were collected on standardized case report forms by site clinical personnel and reviewed by the investigator or qualified study monitors.

Data collected at baseline included limited demographic information, patient medical history, and device and procedural details. In the SECURE study, findings related to complications were collected at the following time points: the first post-operative visit, 4–6 weeks, 3 months following the index procedure, and at an extended follow-up visit just prior to study closure. In the CARE study, data on complications were collected at the following time points: the first post-operative visit, 4–6 weeks, and any other follow-up visits prior to study closure.

### Outcome measures

Clinical outcome measures included the incidence rates of pocket infection, superficial cellulitis, superficial surgical site infection, hematoma, lead dislodgement, and other complications (all collected outcome measures can be found in Supplementary Table 1). A major CIED infection was defined as infection requiring surgical intervention (i.e., system removal, pocket revision, etc.) or treatment with long-term antibiotic therapy (if system removal was not possible) to manage one of the following: (1) superficial cellulitis in the region of the CIED pocket with wound dehiscence, erosion, or purulent drainage; (2) deep incisional or organ/space (pocket) surgical site infection; (3) persistent bacteremia; or (4) endocarditis. Minor CIED infections included those that did not meet one or more of the criteria for major infection.

Safety outcomes were determined by analysis of all device-related adverse events. Device-related events were defined as clinical signs, symptoms, or conditions that were deemed by the investigator to be causally related to the implantation or the performance of the envelope. Causality was adjudicated by the investigators as not related, possibly related, or probably related to the implantation procedure or envelope.

### Infection risk factor characterization

Data collected for each patient’s baseline medical history included infection risk factors based on information in previous studies, which identified factors significantly associated with an increased risk for CIED-related infections: oral systemic anticoagulants, chronic steroid use, renal insufficiency, diabetes, peripheral vascular disease, coronary artery disease, chronic obstructive pulmonary disease, obesity, malnutrition, smoking status, congestive heart failure, malignancy, hypertension, the presence of two or more leads, prior device infection, pocket re-entry within 2 weeks of initial implant, and device replacement/revision (3, 8, 11). Numeric scores were calculated for each patient based on the total number of their respective positive infection risk factors. For cohort analysis comparisons, patients with 0 or 1 infection risk factors were grouped together, and patients who had 2 or more (≥2) infection risk factors were grouped together.

### Cohort group definitions

For cohort analysis, patients were grouped by the type of solution used to hydrate their envelope (i.e., saline or saline with one or more antibiotics added). Results are categorized by rehydration solution group and abbreviated throughout the rest of the manuscript as: *Saline* (saline only without any antibiotics), *Gent Only* (saline with gentamicin only), *Any ABX* + *Gent* (saline with gentamicin, potentially with one or more other antibiotics), and *Any ABX* − *Gent* (saline with one or more antibiotics, not including gentamicin) (Box 1). Statistical comparisons were evaluated between the *Saline* and *Gent Only* groups, and *Any ABX* + *Gent* and *Any ABX* − *Gent* groups.

**BOX 1. Patient cohorts referenced in this manuscript, and the respective envelope hydration solutions chosen by physicians in real-world practice.**

**Table:** 

Cohort name	Envelope hydration solution(s) used
Total	All known hydration solutions combined.
Saline	Hydration in saline only without any antibiotics.
Gent Only	Hydration in saline with gentamicin only.
Any ABX + Gent	Hydration in saline with gentamicin, potentially with one or more other antibiotics.
Any ABX − Gent	Hydration in saline with one or more antibiotics, not including gentamicin.

### Use of preoperative intravenous antibiotic prophylaxis (IV ABX)

Data was collected on the real-world use (and non-use) of IV ABX within the standardized case report forms used for both studies. The rate of IV ABX compliance was determined for each study site by totaling all patients who received IV ABX during their procedure and dividing that number by the total number of patients enrolled by that site. Sites were then grouped into categories of IV ABX compliance: 100% compliance, ≥80% compliance, and <80% compliance.

### Statistical analysis

Continuous variables were assessed for normality. The cohort was then described using means with standard deviations for continuous variables and counts with percentages for categorical variables. Independent samples *t*-tests were used to compare mean differences between groups. Categorical variables were compared using Pearson chi-square tests for comparisons with expected cell counts ≥5. Fisher’s exact tests were reported if ≥1 expected cell count was <5. *P*-values were considered statistically significant if <0.05. To account for familywise error, the significance threshold can be compared at <0.001. SPSS version 26 (IBM, Armonk, NY, USA) was used for statistical analyses.

## Results

**FIGURE 2:**
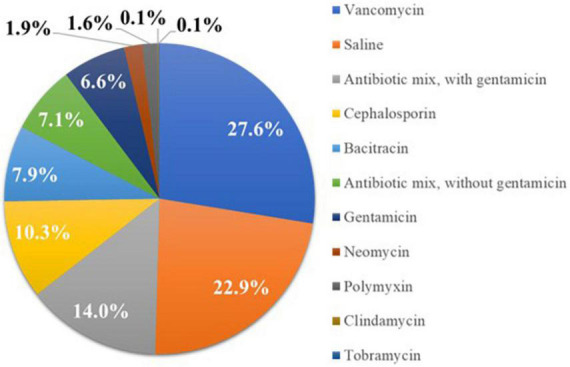
Percentage of patients receiving biologic extracellular matrix (ECM) envelopes hydrated in different solutions prior to implantation. Most implanting physicians chose envelope hydration solutions containing antibiotics.

A total of 1,102 patients (*n* = 94 CARE, *n* = 1,008 SECURE) at 40 centers (*n* = 1: CARE, *n* = 39: SECURE), who received a CIED device implantation using a biologic ECM envelope hydrated in a known solution, were included in the analysis. Nine patients were excluded because the hydration solution used in these cases was unknown. The mean duration of follow-up for the overall sample was 223.7 ± 173.0 days. A total of 252 patients (22.9%) received biologic envelopes hydrated in *Saline*, 73 (6.6%) *Gent Only*, 227 (20.6%) *Any ABX* + *Gent*, and 623 (56.5%) *Any ABX* − *Gent*. The specific antibiotics selected by implanting physicians in the study are illustrated in Figure 2.

### Background characteristics

The demographics and medical histories of enrolled patients are shown in Tables 1, 2. There were no significant differences between subgroups in age (mean 72 years), gender (60.9% male), or mean BMI (28.8 kg/m^2^).

**TABLE 1:** Patient demographics.

Characteristic	Total (*N* = 1,102)	Saline (*n* = 252)	Gent Only (*n* = 73)	Any ABX + Gent (*n* = 227)	Any ABX − Gent (*n* = 623)	*p-value*	
						
						Saline vs. Gent Only	ABX + Gent vs. ABX − Gent
Age, years, mean ± *SD*	72.0 ± 12.0	72.3 ± 11.4	73.6 ± 11.8	72.0 ± 11.7	71.8 ± 12.3	0.406	0.849
Gender, male, *n* (%)	671 (60.9)	145 (57.5)	45 (61.6)	135 (59.5)	391 (62.8)	0.531	0.382
BMI, mean ± *SD*	28.8 ± 6.7	28.6 ± 6.5	28.8 ± 8.5	28.4 ± 7.3	29.1 ± 6.6	0.835	0.719
**Race, *n* (%)**						0.266	**<0.001**
White	874 (79.3)	207 (82.1)	65 (89.0)	205 (90.3)	462 (74.2)	–	–
Black or African American	174 (15.8)	26 (10.3)	7 (9.6)	12 (5.3)	136 (21.8)	–	–
Asian	17 (1.5)	10 (4.0)	0 (0.0)	3 (1.3)	4 (0.6)	–	–
Native Hawaiian or Other Pacific Islander	9 (0.8)	6 (2.4)	0 (0.0)	1 (0.4)	2 (0.3)	–	–
American Indian or Alaska Native	7 (0.6)	0 (0.0)	0 (0.0)	1 (0.4)	6 (1.0)	–	–
Other	11 (1.0)	2 (0.8)	0 (0.0)	4 (1.8)	5 (0.8)	–	–
Unknown	10 (0.9)	1 (0.4)	1 (1.4)	1 (0.4)	8 (1.3)	–	–
**Ethnicity, *n* (%)**						1.00	0.499
Non-hispanic or Latino	1065 (96.6)	249 (98.8)	73 (100.0)	220 (96.9)	596 (95.7)	–	–
Hispanic or Latino	31 (2.8)	0 (0.0)	0 (0.0)	7 (3.1)	24 (3.9)	–	–
Unknown	6 (0.5)	3 (1.2)	0 (0.0)	0 (0.0)	3 (0.5)	–	–

Bolded values are those that have a significant *P*-value.

**TABLE 2:** Patient medical history.

Condition	Total (*N* = 1,102)	Saline (*n* = 252)	Gent Only (*n* = 73)	Any ABX + Gent (*n* = 227)	Any ABX − Gent (*n* = 623)	*p-value*	
						
						Saline vs. Gent Only	ABX + Gent vs. ABX − Gent
Current smoker	128 (11.6)	13 (5.2)	13 (17.8)	23 (10.1)	92 (14.8)	**<0.001**	0.080
CAD	20 (1.8)	0 (0.0)	0 (0.0)	6 (2.6)	14 (2.3)	–	0.736
Diabetes	331 (30.0)	74 (29.4)	10 (13.7)	52 (22.9)	205 (32.9)	**0.007**	**0.005**
Hypertension	45 (4.1)	2 (0.8)	0 (0.0)	3 (1.3)	40 (6.4)	1.00^¥^	**0.003**
Systemic anticoagulant use	273 (24.8)	81 (32.1)	20 (27.4)	68 (30.0)	124 (19.9)	0.440	**0.002**
BMI > 30 kg/m^2^	417 (37.8)	93 (36.9)	24 (32.9)	72 (31.7)	252 (40.4)	0.528	**0.020**
PVD	86 (7.8)	16 (6.3)	5 (6.8)	6 (2.6)	64 (10.3)	0.793^¥^	**<0.001**
CHF/HF	468 (42.5)	84 (33.3)	39 (53.4)	82 (36.1)	302 (48.5)	**0.002**	**0.001**
Chronic steroid use	14 (1.3)	3 (1.2)	0 (0.0)	1 (0.4)	10 (1.6)	1.00^¥^	0.184
COPD	8 (0.7)	0 (0.0)	0 (0.0)	1 (0.4)	7 (1.1)	–	0.362
Malnutrition	12 (1.1)	0 (0.0)	0 (0.0)	3 (1.3)	9 (1.4)	–	0.893
Malignancy	47 (4.3)	12 (4.8)	4 (5.5)	11 (4.8)	24 (3.9)	0.763^¥^	0.519
Renal insufficiency	138 (12.5)	35 (13.9)	7 (9.6)	20 (8.8)	83 (13.3)	0.335	0.075
**Prior device history**						0.992	**<0.001**
PPTP – yes	67 (6.1)	7 (2.8)	2 (2.7)	4 (1.8)	56 (9.0)	–	–
PPTP – unknown	133 (12.1)	3 (1.2)	1 (1.4)	3 (1.3)	127 (20.4)	–	–
Pocket re-entry*	5 (0.5)	0 (0.0)	2 (2.7)	3 (1.3)	2 (0.3)	**0.050**	0.091
Prior device infection**	20 (1.8)	3 (1.2)	0 (0.0)	4 (1.8)	13 (2.1)	1.00	0.765

Values are given as *n*, (%) unless otherwise indicated.

CAD, coronary artery disease; CHF/HF, congestive heart failure/heart failure; COPD, chronic obstructive pulmonary disease; Gent, gentamicin; PVD, peripheral vascular disease; PPTP, pre-procedure temporary pacing.

*Within 2 weeks of initial implant.

**>12 months before current procedure.

^¥^Fisher’s exact test used because ≥1 expected cell count was <5.

Bolded values are those that have a significant *P*-value.

Attributed to the non-randomized design and large sample size, a few significant differences emerged between treatment groups with regard to race, ethnicity, and medical history (Tables 1, 2). There was a significant difference detected for the races treated in the Any ABX + Gent vs. Any ABX − Gent groups (*p* < 0.001) (Table 1). For ethnicity, there were less Hispanic or Latino patients in the Saline group (0.0%).

With regard to medical history, significant differences between treatment groups were found (Table 2). Current smokers were treated more often with Gent Only vs. Saline (*p* < 0.001), yet there was no difference in patients receiving Any ABX + Gent vs. other antibiotics (Gent Only or Any ABX − Gent). Patients with diabetes were preferentially treated with solutions that did not contain gentamicin: either Saline or Any ABX − Gent. Hydration solutions containing antibiotics other than gentamicin were used in patients with hypertension, however, the opposite was true for patients on oral anticoagulants who were treated preferentially with Any ABX + Gent. Any ABX − Gent was chosen significantly more often for patients who were obese (BMI ≥30 kg/m^2^) and those with peripheral vascular disease. Finally, patients with heart failure received Gent Only more frequently than Saline, but when comparing antibiotic solutions physicians preferentially did not choose those containing gentamicin. No significant differences were found between groups for chronic steroid use, COPD, malnutrition, malignancy, or renal insufficiency.

Patients who underwent pre-procedure temporary pacing (PPTP) were more often treated with Any ABX − Gent. However, patients who had a pocket re-entry within 2 weeks of their original implant were preferentially treated with an envelope hydration solution of Gent Only. There were no significant differences in hydration solutions between patients who suffered prior device infections at least 12 months prior to their current procedure.

### Procedure- and device-related factors

Procedure and device-related factors are shown in Table 3. In general, implanting physicians demonstrated a preference for Saline as a hydration solution for low-powered devices (e.g., pacemakers and CRT-P: *p* < 0.001) and an antibiotic solution for high-powered devices (e.g., ICD and CRT-D: *p* < 0.001 vs. Gent Only). Regarding the type of antibiotic hydration used, implanting physicians had a preference for using gentamicin-containing solutions for high-power devices (*p* = 0.004) while there was no antibiotic preference for low-power devices (*p* = 0.137). For re-operations (*p* < 0.001) and capsulectomy procedures (*p* = 0.009), antibiotic hydration was preferred to Saline. Similarly, gentamicin was the preferred antibiotic for re-operation procedures (*p* < 0.001 vs. Saline, *p* = 0.018 vs. Any ABX − Gent) and capsulectomy procedures (*p* = 0.009 vs. Saline, *p* < 0.001 vs. Any ABX − Gent).

**TABLE 3:** Device and procedural details.

Characteristic	Total (*N* = 1,102)	Saline (*n* = 252)	Gent Only (*n* = 73)	Any ABX + Gent (*n* = 227)	Any ABX − Gent (*n* = 623)	*p-value*	
						
						Saline vs. Gent Only	ABX + Gent vs. ABX − Gent
**High vs. low-powered CIED**							
Low powered	549 (49.8)	148 (58.7)	32 (43.8)	101 (44.5)	300 (48.2)	**<0.001**	0.137
Pacemaker	494 (44.8)	140 (55.6)	21 (28.8)	85 (37.4)	269 (43.2)	–	–
CRT-P	55 (5.0)	8 (3.2)	11 (15.1)	16 (7.0)	31 (5.0)	–	–
High powered	505 (45.8)	100 (39.7)	33 (45.2)	111 (48.9)	294 (47.2)	**<0.001**	**0.004**
ICD	260 (23.6)	63 (25.0)	10 (13.7)	41 (18.1)	156 (25.0)	–	–
CRT-D	245 (22.2)	37 (14.7)	23 (31.5)	70 (30.8)	138 (22.2)	–	–
Pocket/lead revision and/or lead replacement	48 (4.4)	4 (1.6)	8 (11.0)	15 (6.6)	29 (4.7)	–	–
**CIED procedure type**							
Re-operative procedure	449 (40.7)	75 (29.8)	48 (65.8)	115 (50.7)	259 (41.6)	**<0.001**	**0.018**
**Procedural information**						**<0.001**	0.274
CIED only	1054 (95.6)	248 (98.4)	65 (89.0)	212 (93.4)	594 (95.4)	–	–
Pocket revision only	14 (1.3)	4 (1.6)	3 (4.1)	3 (1.3)	7 (1.1)	–	–
Lead addition	2 (0.2)	0 (0.0)	0 (0.0)	1 (0.4)	1 (0.2)	–	–
Lead revision only	27 (2.5)	0 (0.0)	5 (6.9)	10 (4.4)	17 (2.7)	–	–
Other*	5 (0.5)	0 (0.0)	0 (0.0)	1 (0.4)	4 (0.6)	–	–
**Capsulectomy performed**	(*n* = 449)	(*n* = 75)	(*n* = 48)	(*n* = 115)	(*n* = 259)	**0.009**	**<0.001**
Yes	213 (47.4)	24 (32.0)	25 (52.1)	81 (70.4)	108 (41.7)	–	–
Unknown	33 (7.3)	5 (6.7)	7 (14.6)	7 (6.1)	21 (8.1)	–	–

Values are given as *n*, (%) unless otherwise indicated.

*Lead replacement only and lead revision/replace, pocket and lead revision, pocket/lead revision + lead replacement.

Bolded values are those that have a significant *P*-value.

### Infection and other adverse events

There were 28 reported hematomas (2.5%), 14 of which required intervention (1.3%). In no case did hematoma and infection co-occur. There were no significant differences between treatment groups for any individual adverse event, including among subgroups (e.g., use of high- vs. low-power devices). There were also 14 (1.3%) other events reported such as lead dislodgement/revision, pocket revision, erosion, and erythema/fever without differences between groups (Table 4).

**TABLE 4:** Adverse events.

Adverse event	Total (*N* = 1,102)	Saline (*n* = 252)	Gent Only (*n* = 73)	Any ABX + Gent (*n* = 227)	Any ABX − Gent (*n* = 623)	*p-value*	
						
						Saline vs. Gent Only	ABX + Gent vs. ABX − Gent
**Infection**							
Major CIED infection	19 (1.7)	5 (2.0)	1 (1.4)	1 (0.4)	13 (2.1)	1.00^¥^	0.129^¥^
Pocket infection^†^	12 (1.1)	2 (0.8)	0 (0.0)	0 (0.0)	10 (1.6)	1.00^¥^	0.070^¥^
Superficial cellulitis*	7 (0.6)	3 (1.2)	1 (1.4)	1 (0.4)	3 (0.5)	1.00^¥^	1.00
Bacteremia or endocarditis^†^	1 (0.1)	0 (0.0)	0 (0.0)	0 (0.0)	1 (0.2)	–	1.00^¥^
Minor CIED infection**	15 (1.4)	4 (1.6)	2 (2.7)	2 (0.9)	9 (1.4)	0.620^¥^	0.737^¥^
Infections 0–30 days	(*n* = 1,102)	(*n* = 252)	(*n* = 73)	(*n* = 227)	(*n* = 623)	–	–
Major	11 (1.0)	1 (0.4)	1 (1.4)	1 (0.4)	9 (1.4)	0.399^¥^	0.304^¥^
Pocket	7 (0.6)	1 (0.4)	0 (0.0)	0 (0.0)	6 (1.0)	1.00^¥^	0.351^¥^
Infections 31–90 days	(*n* = 1,020)	(*n* = 238)	(*n* = 70)	(*n* = 210)	(*n* = 572)	–	–
Major	8 (0.8)	4 (1.7)	0 (0.0)	0 (0.0)	4 (0.7)	0.578^¥^	0.579^¥^
Pocket	5 (0.5)	1 (0.4)	0 (0.0)	0 (0.0)	4 (0.7)	1.00^¥^	0.579^¥^
**Hematoma**							
Total sample	28 (2.5)	9 (3.6)	1 (1.4)	5 (2.2)	14 (2.2)	0.467^¥^	0.969
Hematoma requiring intervention	14 (1.3)	3 (1.2)	1 (1.4)	4 (1.8)	7 (1.1)	1.00^¥^	0.496^¥^
**Other adverse events**							
Lead dislodgement/revision	3 (0.3)	0 (0.0)	0 (0.0)	1 (0.4)	2 (0.3)	–	1.00^¥^
Pocket revision	6 (0.5)	1 (0.4)	1 (1.4)	2 (0.9)	3 (0.5)	0.399^¥^	0.614^¥^
Erosion	2 (0.2)	2 (0.8)	0 (0.0)	0 (0.0)	0 (0.0)	1.00^¥^	–
Erythema/fever	3 (0.3)	0 (0.0)	0 (0.0)	0 (0.0)	3 (0.5)	–	0.569^¥^

Values are given as *n*, (%) unless otherwise indicated.

*Superficial cellulitis with dehiscence, erosion, or purulent drainage.

**Infections that did not meet one or more of the criteria for major infection.

^¥^Fisher’s exact test used because ≥1 expected cell count was <5.

^†^One subject had pocket infection, bacteremia, and endocarditis.

Overall, there were 19 major CIED infections (1.7%) and 15 minor CIED infections (1.4%) (Table 4). Major infections included 12 pocket infections (1.1%) and 7 instances of superficial cellulitis with dehiscence, erosion, or purulent drainage (0.6%). One patient had bacteremia, endocarditis, and pocket infection. Overall, there were no significant differences detected between the groups. Analysis of time to onset of infection also did not identify any significant differences among treatment groups for major, minor, or pocket infections at any timepoint through 90 days (0–30 days or 31–90 days, Table 4). However, across all timepoints, there was a trend toward statistical significance in the incidence of pocket infections between Any ABX + Gent (0 pocket infections [0%]) and Any ABX − Gent (10 pocket infections [1.6%]) (*p* = 0.070), suggesting a potential benefit to the use of gentamicin for the prevention of pocket infections.

A multivariate logistic regression model was used to predict the likelihood of infection (either major or minor) from the use of gentamicin while adjusting for the risk factors of obesity and diabetes as demonstrated in Table 5. Covariates in this model were statistically significant, including a history of obesity (odds ratio [OR] 2.3, 95% CI, 1.0 – 5.1) and diabetes (OR 0.2, 95% CI, 0.1 – 0.8). A group difference emerged at the trend level (*p* = *0.083*) such that the omission of gentamicin was associated with a threefold increase in risk for infection (OR 3.0, 95% CI, 1.0 – 10.0). The model area under the curve (AUC) was 0.70 (95% CI, 0.60 – 0.78).

**TABLE 5:** Summary of logistic regression model predicting the presence of any major or minor infection.

	Odds ratio (95% CI)	*p-value*
Obesity (BMI > 30 kg/m^2^)	2.3 (1.0–5.1)	**0.049**
Diabetes	0.2 (0.1–0.8)	**0.018**
No gentamicin	3.0 (1.0–10.0)	0.083
Model AUC	0.70 (0.60–0.78)	

Bolded values are those that have a significant *P*-value.

When subjects were grouped by number of standard infection risk factors (0–9), 88.7% had at least 1 risk factor, and the majority of patients (58.6%) had 2 or more (Table 6). Significantly more patients with 2 or more (≥2) risk factors were in the Gent Only vs. Saline group (*p* = 0.026) or Any ABX − Gent group vs. Any ABX + Gent group (*p* = 0.035).

**TABLE 6:** Analysis by number of infection risk factors by treatment group.

No. infection risk factors	Total (*N* = 1,102)	Saline (*n* = 252)	Gent Only (*n* = 73)	Any ABX + Gent (*n* = 227)	Any ABX − Gent (*n* = 623)	*p-value*	
						
						Saline vs. Gent Only	ABX + Gent vs. ABX − Gent
0–1	456 (41.4)	120 (47.6)	24 (32.9)	103 (45.4)	233 (37.4)	**0.026**	**0.035**
≥2	646 (58.6)	132 (52.4)	49 (67.1)	124 (54.6)	390 (62.6)		

Values are given as *n*, (%) unless otherwise indicated.

Bolded values are those that have a significant *P*-value.

### Use of preoperative intravenous antibiotic prophylaxis (IV ABX)

Overall compliance with IV ABX across all study sites was 96.6% (range 11–100%), similar to WRAP-IT (94.2%), (21) but varied by site: 23 sites (58%) administered IV ABX 100% of the time, 32 sites (80%) administered ≥90% of the time, and 36 sites (90%) administered ≥80% of the time. Only 4 sites (10%) used IV ABX less than 80% of the time.

**FIGURE 3:**
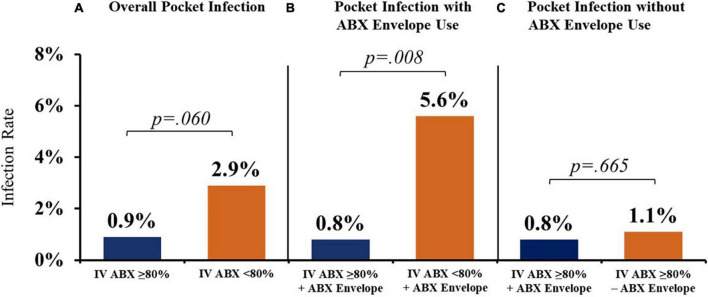
Cardiac implantable electronic device (CIED) pocket infection rates with various prevention strategies. **(A)** Higher IV ABX compliance (≥80% use) was associated with a lower overall rate of CIED pocket infection vs. sites with <80% IV ABX compliance. **(B)** When stratifying for antibacterial biologic envelope usage in conjunction with IV ABX ≥80% site compliance vs. <80% compliance, there was a significant difference in pocket infection rates. **(C)** Use of an antibacterial (vs. saline-only) envelope was associated with a trend toward a lower infection rate for sites with IV ABX compliance ≥80%. IV ABX, pre-operative IV antibacterial prophylaxis; ABX envelope, biologic envelope hydrated with antibiotic solution.

Sites with higher IV ABX compliance (≥80% use) demonstrated a trend toward a lower overall rate of CIED pocket infection than sites with <80% IV ABX compliance (0.9% vs. 2.9%) (Figure 3A). These differences were significantly more pronounced when stratifying for antibacterial biologic envelope usage in conjunction with IV ABX ≥80% site compliance vs. <80% compliance (0.8% vs. 5.6%) (Figure 3B). For sites with IV ABX compliance ≥80%, the use of an antibacterial vs. saline-only hydration envelope was associated with a trend toward a lower infection rate (0.8% vs. 1.1%) (Figure 3C).

Patients who received antibacterial biologic envelopes had a significantly higher average number of infection RFs compared to patients who received saline-hydrated biologic envelopes (2.1 vs. 1.7, *p* < 0.001). Similarly, sites with higher IV ABX compliance (≥80%) tended to treat patients with slightly higher average infection RFs vs. sites that used IV ABX on <80% of their patients (2.0 vs. 1.8, *p* = 0.077).

## Discussion

This study represents the largest dataset of the biologic envelope usage to date. Real-world clinical decision-making regarding the choice of hydration solution for biologic CIED envelopes varies among implanting physicians. Determining how physicians make these decisions and how their choices impact clinical outcomes is important to identify best practices. In the current analysis, the use of biologic envelopes hydrated in antibiotic solutions containing gentamicin generally was associated with reduced risk for infection compared to antibiotic solutions not containing gentamicin. Many implanting physicians seemed to perceive the use of gentamicin as beneficial in limiting infection risk as they tended to select solutions containing gentamicin for patients with the highest infection risk.

The overall rate of infection through 90 days in this analysis was 3.1%, divided closely among major (1.7%) and minor (1.4%) CIED infections. This incidence of infection is consistent with the low rate of infection previously reported in the WRAP-IT trial, (21) and other literature for *de novo* placements (∼1–3%), (2–4) particularly when considering the high-risk status of most patients in this study and the randomized trial. Most subjects (58.6%) in this study had 2 or more infection risk factors. Patients were, on average, overweight (mean BMI 28.8 kg/m^2^, 37.8% obese), nearly half had heart failure (42.5%), about one-third had diabetes (30%), one quarter used systemic anticoagulants (24.8%), and many had renal insufficiency (12.5%) or were current smokers (11.6%). For comparison, the WRAP-IT trial enrolled Envelope subjects with similar infection risk factors: mean BMI was 29.1 kg/m^2^, 68% had cardiomyopathy, 31% had diabetes, 39.5% were on anticoagulants, and 16.8% had renal dysfunction (21). Smoking status was not reported by the authors.

Multiple studies have demonstrated variation in infection rates based on procedure- and patient-related factors, including *de novo* placements vs. reoperations, the type of device implanted (e.g., pacemaker vs. CRT-D), patient comorbidities, and the use of antibiotic therapy or antibiotic eluting envelopes (37). The prospective WRAP-IT and PADIT studies, which intentionally enrolled patients with high infection risk, demonstrated that low infection rates (∼1% major infections) can be achieved in complex procedures and patients through the use of evidence-based approaches, such as the use of incremental perioperative antibiotics or antibiotic eluting envelopes (19, 21).

In our study, over 40% of the subjects were undergoing reoperation for device replacement, a procedure that is associated with infection rates ranging up to 7% in previous studies (2–5). Indeed, implanting surgeons showed a preference for hydration solutions that included antibiotics, particularly gentamicin, for patients undergoing reoperation, regardless of device type (Table 3). With regard to type of device, there was a preference for gentamicin-containing solutions when surgeons implanted high- vs. low-power devices, a finding consistent with studies that have identified complex devices as risk factors for CIED infection (2–4, 11).

Interestingly, there were no significant differences between groups with regard to patients with prior device infections (Table 2). The majority of this group received biologic envelopes hydrated in Any ABX − Gent (*n* = 13). There was a preference for gentamicin over saline among the few patients undergoing pocket re-entry (*p* = 0.050). However, the total number of patients in these subgroups was small (*n* = 20 total prior device infections, 1.8%; *n* = 5 pocket re-entry, 0.5%), limiting further interpretation of these outcomes.

In our study population, the presence of multiple infection risk factors was significantly associated with the use of antibiotics in the hydration solution: Gent Only was chosen over Saline, and Any ABX − Gent was chosen over Any ABX + Gent. Based on this analysis, the threshold at which surgeons demonstrated a preference for envelope hydration in antibiotics is 2 or more risk factors. The results of the logistic regression modeling further support the use of gentamicin in high-risk patients. This adjusted model found that the omission of gentamicin from the CIED hydration solution was associated with a threefold increase in risk for infection (OR 3.0, 95% CI, 1.0 – 10.0), although it did not reach statistical significance (*p* = 0.083). The AUC was 0.70 (95% CI, 0.60 – 0.78), which suggests good discriminative ability.

Although both our study and the WRAP-IT trial (21) enrolled subjects with similar clinical profiles and resulted in similar outcomes, there are obvious design differences between both studies which allow us the opportunity to continue improving clinical outcomes by expanding our knowledge on device envelopes and their appropriate use. The purpose of this study was to observe physician practice patterns during real-world usage of the biologic envelope, so patients were not randomized. In our study, physicians could enroll subjects receiving any brand of CIED with the biologic envelope, which supports the safety and efficacy of antibiotic-eluting envelopes when used with various manufacturer CIEDs, as also found in previous smaller studies (9, 13, 17, 36, 38). Finally, we looked closely at preoperative antibiotic prophylaxis compliance (which similar to the WRAP-IT trial was not controlled in our study) in conjunction with envelope usage, which revealed diverse and some concerning practice patterns. This finding is also corroborated by a recent independent survey of implanting physicians in the USA, which is further described below (39).

### CIED envelopes and choice of antibiotics

Previous studies have established the efficacy of antibiotic eluting CIED envelopes for reducing infection risk, particularly in high-risk patients (21, 40–42). Meta-analyses of published studies report >60% reductions in major CIED infections with these devices (40–42). Options for antibiotic envelopes include the previously described non-biologic envelope impregnated with rifampin and minocycline or hydration of the biologic CanGaroo Envelope in saline containing one or more antibiotics. Surgeons in the current study used a wide variety of antibiotics, often in combination (Figure 2). No subjects in our database received a hydration solution containing rifampin and/or minocycline. However, this finding may have been due to limited availability of these antibiotics in the OR. Because potential pathogens can vary by patient and hospital, there may be advantages to allowing implanting physicians to select antibiotics based on their knowledge of local and patient factors.

Our study outcomes suggest potential advantages to the use of gentamicin in high-risk patients. Gentamicin is an aminoglycoside with broad-spectrum bactericidal activity, including against *Staphylococcus* species, which are the most commonly identified pathogens in CIED infections, (43) and aerobic Gram-negative organisms. The main clinical limitation of gentamicin is its association with risks for nephrotoxicity and ototoxicity when administered systemically (44). Conversely, local administration of gentamicin has demonstrated efficacy in multiple surgical indications, including preventing CIED-related infections, without the risks of systemic exposure (42, 45–48).

Preclinical studies indicate that biologic CIED envelopes soaked in gentamicin can deliver high concentrations of gentamicin to the surrounding tissues, with minimal systemic exposure, providing excellent efficacy against *Staphylococcus* spp. and other CIED pathogens (42, 45, 49). One preclinical study, which compared biologic envelopes soaked in gentamicin, vancomycin, or both, found greater *in vitro* antimicrobial activity with gentamicin compared to vancomycin (49). A retrospective clinical study of 1266 consecutive patients undergoing CIED replacements reported no CIED-related infections requiring device extraction with the use of a gentamicin-soaked collagen sponge over a mean of 3.5 years of follow-up (4,285 patient-years), even in this high-risk population (45). Data from other cardiac surgical procedures also support the efficacy of local gentamicin delivery using ECM or collagen sponges to prevent wound infections (47, 48, 50).

### ECM biomaterials and infection risk

Compared with non-biologic biomaterials, such as those used in other types of CIED envelopes, biomaterials made from biologic non-crosslinked ECM, such as CanGaroo, have been shown to foster greater tissue integration and vascular ingrowth, a reduced inflammatory response, and more rapid clearance of bacteria (22, 23, 25–31). Because of these characteristics, biologic ECM envelopes may be preferred for higher-risk patients, such as those in the current analysis. Indeed, two recent studies of patient characteristics associated with the use of a biologic envelope identified a preference for these devices in patients who were elderly and had poor tissue quality, had a history of prior device infection, or had major infection risk factors (36, 38).

Hydration of biologic envelopes in antibiotic solutions has the additional advantage of providing antibiotics where they are most needed. In preclinical studies, biologic envelopes hydrated in antibiotic solutions showed a biphasic pattern of antibiotic release, with an initial bolus followed by sustained release over several days (49). Because CIED infections presumably occur at the time of implantation, high and sustained local antibiotic concentrations should be ideal for infection prevention. As shown clinically in a randomized controlled trial, antibiotic-eluting envelopes can have a sustained effect on lowering infection risk in CIED patients (21).

As noted, the current study included high proportions of patients with multiple infection risk factors (58.6%), receiving high-powered devices (45.8%), and undergoing re-operation (40.7%). Despite these high-risk features, the overall infection rate was modest at 3.1%, with about half being major infections (1.7%). Although this incidence is based on a relatively modest follow-up period (mean 224 ± 173.0 days, with no infections reported after 102 days), and the total number would be expected to increase marginally with longer follow-up (51, 52), these initial findings suggest that surgeons’ use of antibacterial biologic envelopes (particularly containing gentamicin) may have reduced the risk of infection. Further reductions might be achieved with wider use of gentamicin, possibly in addition to other antibiotics, when hydrating the biologic envelopes prior to implantation, and proper employment of IV ABX. Based on the data discussed above, the CanGaroo Envelope was recently cleared in the E.U. for hydration in 20 mL of gentamicin (40 mg/mL) prior to implantation, although this hydration solution is not currently cleared for use in the U.S. (53).

### Infection risk mitigation should be a multi-pronged approach

Observation of physician decision-making with real-world usage of antibacterial envelopes during CIED implantations demonstrated variable usage of pre-operative IV antibacterial prophylaxis (IV ABX). Across all sites, the overall rate of IV ABX compliance in our real-world study was 96.6%. However, a concerning number of patients undergoing CIED implantation did not receive guideline-recommended IV ABX and this group had a higher infection rate. Our findings are similar to recently published results from an independent survey of antibiotic use during CIED implantation in the United States. Although the survey respondents reported a 97% rate of routine use of pre-operative systemic antibiotics (similar to our 96.6% overall compliance rate), the authors also found that there were wide variations in implanter practices (39).

Even in a randomized, controlled trial evaluating the infection risk reduction of a non-biologic antibacterial envelope, only 94.2% of study sites followed guideline-recommended IV ABX (21). In our study tracking real-world physician practice, the rate of IV ABX compliance was only slightly higher. Most sites (90%) administered IV ABX to ≥80% of their patients, yet only 80% of sites administered IV ABX to their patients ≥90% of the time. Only about half (58%) of sites in this study employed IV ABX 100% of the time. Surprisingly, 10% of sites used IV ABX <80% of the time.

Sites employing IV ABX ≥80% of the time had a lower overall rate of CIED pocket infection than sites with <80% IV ABX compliance (0.9% vs. 2.9%), which was significantly more pronounced when antibacterial envelopes were used alongside IV ABX (0.8% vs. 5.6%) (Figures 3A,B). The patients who received antibacterial envelopes had a significantly higher average number of infection RFs compared to patients who received saline-hydrated biologic envelopes, yet for sites with IV ABX compliance ≥80% who hydrated the biologic envelope in an antibacterial vs. saline-only hydration solution, the use of an antibacterial envelope was associated with a trend toward a lower infection rate (0.8% vs. 1.1%) (Figure 3C). Thus, patients with higher infection risk were more frequently receiving infection prevention therapies compared to lower-risk patients.

Considering the use of IV ABX falls under the guideline recommendations, it is unclear the rational for not using IV ABX on every patient. Three potential reasons to explain the discrepancy could be that: (1) some physicians are following outdated practice or institutional standards, (2) there is a false sense of security when using antibacterial envelopes that IV ABX is not needed in conjunction, or (3) when treating patients with lower infection risk, IV ABX is not considered as often. Our observations align with the current guideline recommendations which recommend IV ABX use during 100% of CIED implantations (18). These findings suggest that the use of antibacterial envelopes without adjunct IV ABX is not sufficient to reduce CIED infections.

## Limitations

The major limitations of this study are its non-randomized design, limited duration of follow-up, and single-arm design. The lack of randomization allowed for bias in the selection of enrolled patients for implantation with biologic envelopes, institutional policies, or physician standard of care for use of guideline-recommended IV ABX, and in the decision to use or not to use specific antibiotics or antibiotic combinations for envelope hydration. Because of the lack of randomization and the single-arm design, which prevented comparisons with other treatment approaches, the intention of this analysis was to describe real-world surgeon practice patterns. Although the overall sample was relatively large (*N* = 1,102), only about 20% of cases included the use of gentamicin (*n* = 227). Finally, the duration of follow-up may not have captured late adverse events, limiting data on long-term efficacy of the biologic envelope.

Significant differences were identified between treatment groups with regard to race and ethnicity. These differences may reflect surgeon choices based on underlying infection risk factors in these subgroups. Alternatively, they may suggest treatment bias based on race and ethnicity. These possibilities should be addressed in future studies that are specifically designed to analyze infection risk and treatment decisions based on race and ethnicity.

## Conclusion

The results of this analysis provide further evidence that biologic CIED envelopes are associated with low infection risk, especially when combined with guideline-recommended intravenous antibacterial prophylaxis. In this high-risk population, the use of a biologic envelope led to a low rate of major infections (<2%). The results further suggest that hydration of the biologic envelope in antibiotic-containing solutions, particularly gentamicin, may help to reduce infection risk. Allowing implanting physicians to select appropriate antibiotics during rehydration may have advantages in targeting antimicrobial therapy to local and patient-specific factors. Larger studies are needed to better understand these potential benefits and define the clinical role of antibiotic selection for CIED envelope hydration.

## Data availability statement

The raw data supporting the conclusions of this article will be made available by the authors, without undue reservation.

## Ethics statement

The studies involving human participants were reviewed and approved by WIRB-Copernicus Group (WCG^®^ IRB). The patients/participants provided their written informed consent to participate in this study.

## Author contributions

TD substantially contributed to the drafting of the manuscript. TD and DW substantially contributed to the conception, design, data collection, and analysis of the work. JC and DW contributed their expertise to the analysis and interpretation of data and to reviewing and editing the manuscript. All authors accept accountability for the accuracy of this work, and drafted, revised, and approved the final version of the manuscript to be published within this journal.
