# Supplementary material for: Physician antibiotic hydration preferences for biologic antibacterial envelopes during cardiac implantable device procedures
Source: Front Cardiovasc Med. 2022 Dec 22;9:1006091. doi: 10.3389/fcvm.2022.1006091 (PMC9815182; doi:10.3389/fcvm.2022.1006091)
Supplement: Supplementary file 1 [file Table_1.DOCX]

**Supplementary Table 1.** All Clinical Outcome Measures Collected

| Urinary tract infection  Cellulitis at IV site  Superficial wound healing  Myocardial infarction  Incisional bleeding  Ecchymosis  Gout  Phychiatric  Fever  Bronchiectosis  Vertigo  Musculoskeletal pain  Syncope  Cholelithiasis  Lead dislodgement/revision  Chest pain  Exacerbation of COPD  Renal failure  Pleural effusion  Inappropriate shocks  Unrelated trauma  Incision revision  Arrhythmia  Device reprogramming  Dehiscence  Erythema  Allergic reaction (adhesive)  Exacerbation of heart failure  Pneumonia  Pocket revision  Infection  Bruising  Seroma  Death  Hematoma  Discomfort  Swelling  Erosion |
| --- |

COPD, chronic obstructive pulmonary disease; IV, intravenous
